# Molecular pharmacodynamics of meropenem for nosocomial pneumonia caused by *Pseudomonas aeruginosa*

**DOI:** 10.1128/mbio.03165-23

**Published:** 2024-01-18

**Authors:** Nicola Farrington, Vineet Dubey, Adam Johnson, Iona Horner, Adam Stevenson, Jennifer Unsworth, Ana Jimenez-Valverde, Julie Schwartz, Shampa Das, William Hope, Christopher A. Darlow

**Affiliations:** 1 Antimicrobial Pharmacodynamics and Therapeutics, Department of Pharmacology, University of Liverpool, Liverpool Health Partners, Liverpool, United Kingdom; 2 Charles River Laboratories, Reno, Nevada, USA; McMaster University, Hamilton, Ontario, Canada

**Keywords:** meropenem, animal models, antimicrobial combinations, antimicrobial resistance, mathematical modeling, molecular biology, *Pseudomonas aeruginosa*, rabbit, carbapenems, pneumonia, gene sequencing, mechanisms of resistance

## Abstract

Hospital-acquired pneumonia (HAP) is a leading cause of morbidity and mortality, commonly caused by *Pseudomonas aeruginosa*. Meropenem is a commonly used therapeutic agent, although emergent resistance occurs during treatment. We used a rabbit HAP infection model to assess the bacterial kill and resistance pharmacodynamics of meropenem. Meropenem 5 mg/kg administered subcutaneously (s.c.) q8h (±amikacin 3.33–5 mg/kg q8h administered intravenously[i.v.]) or meropenem 30 mg/kg s.c. q8h regimens were assessed in a rabbit lung infection model infected with *P. aeruginosa*, with bacterial quantification and phenotypic/genotypic characterization of emergent resistant isolates. The pharmacokinetic/pharmacodynamic output was fitted to a mathematical model, and human-like regimens were simulated to predict outcomes in a clinical context. Increasing meropenem monotherapy demonstrated a dose-response effect to bacterial kill and an inverted U relationship with emergent resistance. The addition of amikacin to meropenem suppressed the emergence of resistance. A network of porin loss, efflux upregulation, and increased expression of AmpC was identified as the mechanism of this emergent resistance. A bridging simulation using human pharmacokinetics identified meropenem 2 g i.v. q8h as the licensed clinical regimen most likely to suppress resistance. We demonstrate an innovative experimental platform to phenotypically and genotypically characterize bacterial emergent resistance pharmacodynamics in HAP. For meropenem, we have demonstrated the risk of resistance emergence during therapy and identified two mitigating strategies: (i) regimen intensification and (ii) use of combination therapy. This platform will allow pre-clinical assessment of emergent resistance risk during treatment of HAP for other antimicrobials, to allow construction of clinical regimens that mitigate this risk.

**IMPORTANCE:**

The emergence of antimicrobial resistance (AMR) during antimicrobial treatment for hospital-acquired pneumonia (HAP) is a well-documented problem (particularly in pneumonia caused by *Pseudomonas aeruginosa*) that contributes to the wider global antimicrobial resistance crisis. During drug development, regimens are typically determined by their sufficiency to achieve bactericidal effect. Prevention of the emergence of resistance pharmacodynamics is usually not characterized or used to determine the regimen. The innovative experimental platform described here allows characterization of the emergence of AMR during the treatment of HAP and the development of strategies to mitigate this. We have demonstrated this specifically for meropenem—a broad-spectrum antibiotic commonly used to treat HAP. We have characterized the antimicrobial resistance pharmacodynamics of meropenem when used to treat HAP, caused by initially meropenem-susceptible *P. aeruginosa*, phenotypically and genotypically. We have also shown that intensifying the regimen and using combination therapy are both strategies that can both treat HAP and suppress the emergence of resistance.

## INTRODUCTION

Hospital-acquired (or nosocomial) pneumonia (HAP) is a leading cause of morbidity and mortality (1, 2). The causative pathogens typically include *Staphylococcus aureus* and Gram-negative bacilli (particularly Enterobacterales, *Pseudomonas aeruginosa*, and *Acinetobacter baumannii*) as the most prevalent pathogens (3). Escalating antimicrobial resistance (AMR) is a globally important problem (4). The high bacterial densities that are characteristic of HAP, which are typically multiples of the inverse of mutational frequency of resistance (indicating that pre-existing resistant mutants will likely be present within the inoculum), mean that the emergence of resistance on therapy is the norm (5, 6). Mitigation requires a detailed understanding of the molecular pharmacodynamics of bacterial killing and the emergence of resistance.

Meropenem is a carbapenem with potent microbiological activity against *P. aeruginosa* (7, 8). Its activity against multiple drug-resistant (MDR) pathogens, its demonstrated efficacy for pneumonia, and its extensive safety databases mean it is a standard-of-care for HAP (1, 9–13). Nevertheless, *P. aeruginosa* develops resistance to meropenem via a plethora of molecular mechanisms that includes porin modification, drug efflux, and ß-lactamase-related hydrolysis (14, 15). The emergence of resistance in *P. aeruginosa* has been observed on therapy (16, 17), and this potentially results in clinical failure and subsequent horizontal spread of resistant isolates, affecting distant others.

Laboratory animal models of hospital-acquired pneumonia have been used previously in drug development to define optimal antimicrobial regimens (18). The pharmacodynamics of the emergence of resistance is usually estimated in hollow-fiber infection models (HFIM), where high microbial densities that mimic those observed in HAP can be studied (19, 20). These inocula typical of HAP/VAP are generally lethal in mice, making standard murine models difficult to use for work examining development of resistance. However, there are important limitations of HFIM, which may include the following: (i) avid binding to plastic (ii), absence of anatomical barriers and multiple pulmonary subcompartments relevant to pathogenesis of HAP/VAP (e.g., pulmonary alveolar macrophages and alveolar capillary barrier), and (iii) absence of immunological effectors central to the pathogenesis and clinical response of HAP. Moreover, nosocomial pneumonia remains an extremely challenging clinical entity with many examples of failed clinical studies of new antimicrobial agents (21–23). Hence, new experimental platforms are required that enable effective and resilient regimens for pneumonia to appropriately design and de-risk clinical trials for HAP (24), which are typically difficult and expensive.

Here, we address this issue by developing and characterizing a novel rabbit infection model of HAP to simultaneously assess antibacterial killing and the emergence of resistance. Unlike the murine model, the rabbit model allows serial pharmacokinetic sampling and can tolerate higher inocula for longer, potentially allowing observation of the emergence of resistance. Meropenem was studied given the primacy of this agent for HAP—it serves as a benchmark for the assessment of novel antimicrobial agents that are being considered for development as agents for HAP.

(The results of this work were presented, in part, at the 2023 ASM/ESCMID Joint Conference on Drug Development to Meet the Challenge of Antimicrobial Resistance, 19–22 September 2023, in Boston, MA.)

## RESULTS

### Challenge strain and MIC

*Pseudomonas aeruginosa* ATCC 27853 was used as the challenge strain throughout. The MIC of meropenem and amikacin was 1 mg/L and 2 mg/L, respectively, using European Committee on Antimicrobial Susceptibility Testing (EUCAST) broth microdilution methodology (25).

### Development of an experimental rabbit model of hospital-acquired pneumonia

Preliminary experiments were performed to establish experimental conditions to enable a lethal model that provided reproducible readouts. The immunosuppression regimen, duration of experiment, endobronchial inoculum, and timing of antimicrobial initiation post inoculation were investigated. The final study design was a 96-hour model (time = 0 is the time of inoculation) using a target inoculum of 5 × 10^7^ CFU/mL. A total of 2 mL was inoculated into the endobronchial tree to deliver 1 × 10^8^ CFU. Antimicrobial therapy commenced 24 hours post infection. Animals received cytarabine 525 mg/m^2^ q24h, initiated 24 hours prior to inoculation.

### Pharmacodynamics of meropenem and amikacin

**Fig 1:**
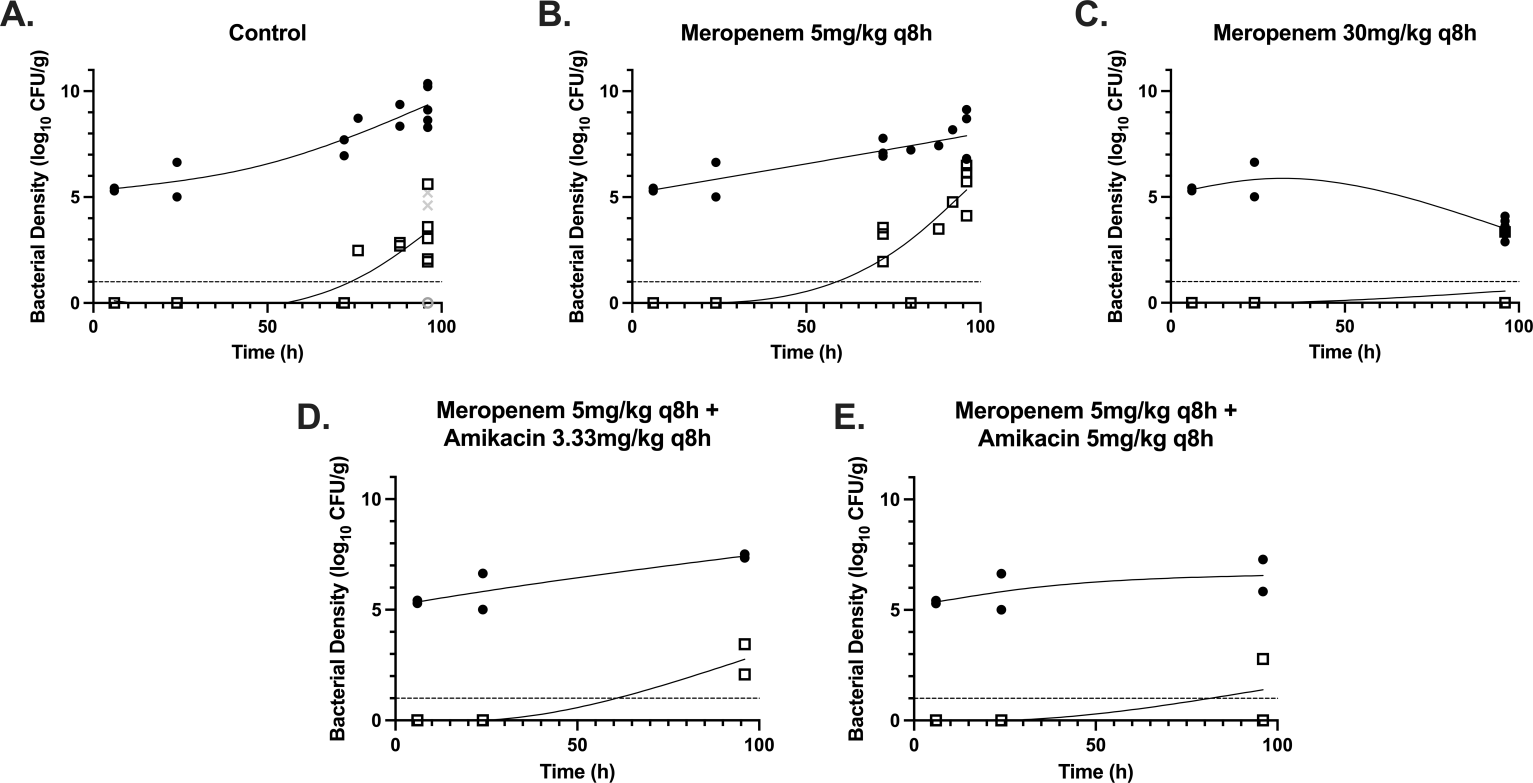
(A–E) Pharmacodynamics of no-treatment control (*n* = 14) meropenem 5 mg/kg q8h (*n* = 14) and 30 mg/kg q8h (*n* = 10) s.c. as monotherapy and meropenem 5 mg/kg q8h in combination with amikacin 3.33 mg/kg q8h adminstered intravenously (i.v.) (*n* = 6) or 5 mg/kg qh i.v. (*n* = 6) assessed using the bacterial density per gram of lung tissue. Filled circles depict total bacterial counts; open squares depict resistant bacterial counts. Treatment was initiated at 24 hours. The solid black lines are LOWESS curves fitted to the total and resistant bacterial densities in each cohort of rabbits. The dashed line indicates the limit of detection. The gray Xs and circles depict the total and resistant bacterial counts from two control rabbits, whose bacterial inoculum failed to expand. They are shown here for completeness but were not included in any analysis or the no-treatment control total numbers.

**Fig 2:**
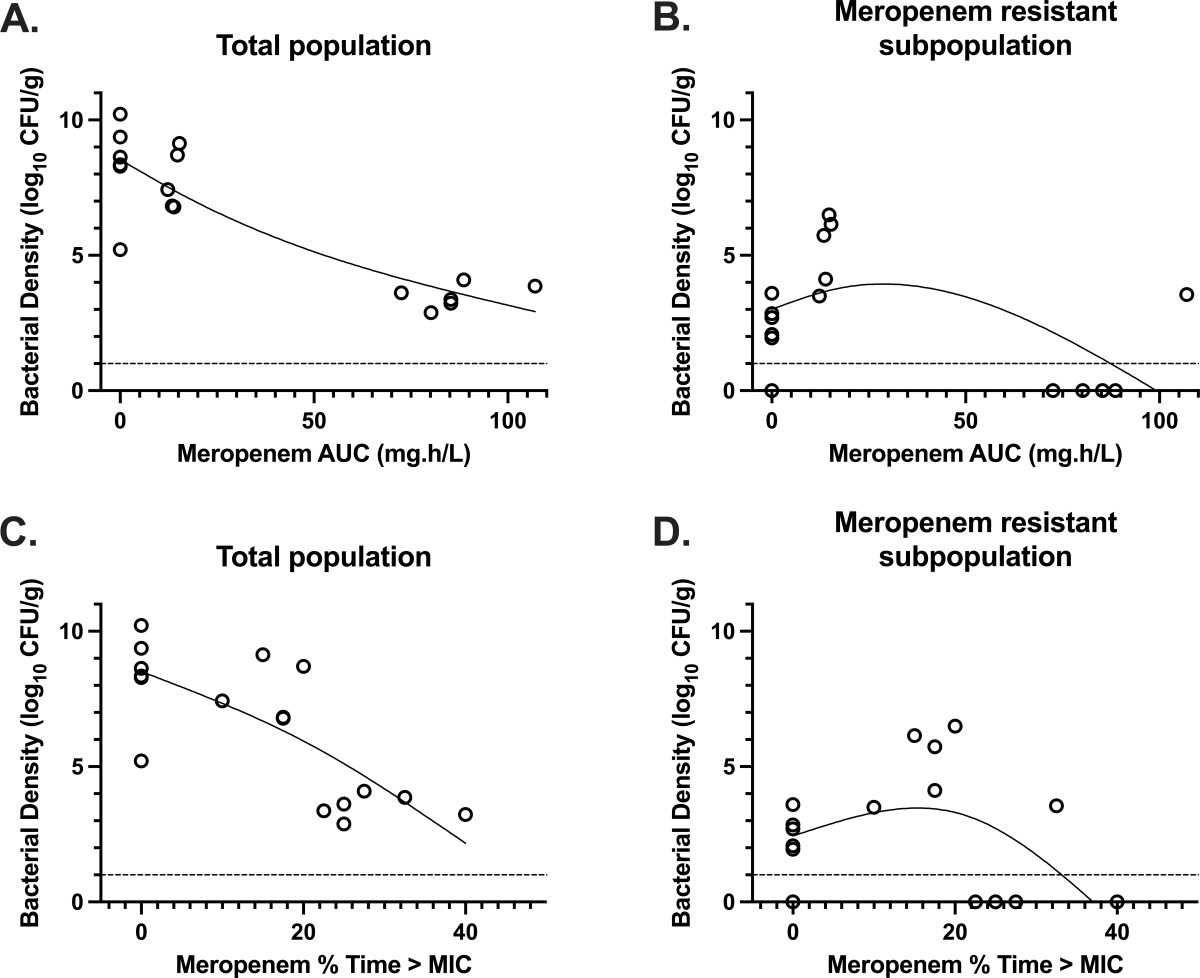
End-experiment total population (A and C) and meropenem-resistant subpopulation (B and D) bacterial densities in lung tissue by exposure of meropenem by AUC (A and B) and % time above MIC (C and D) — both determined from individual Bayesian posteriors from the pharmacokinetic-pharmacodynamic (PK-PD) modeling over the 24-hour period following first administration for the free fraction. Only data points obtained in the last dosing interval of the experiment (i.e., 88–96 hours) are included. The solid black lines are LOWESS curves fitted to the total and resistant bacterial density data. The dashed lines indicates the limit of detection.

Rabbits received vehicle control (*n* = 14), meropenem 5 mg/kg administered subcutaneously (s.c.) q8h (*n* = 14), or meropenem 30 mg/kg s.c. q8h (*n* = 10) 24 hours following inoculation (Fig. 1). There was a dose-dependent reduction in bacterial density (Fig. 2A and C). The emergence of meropenem-resistant bacteria increased in rabbits receiving meropenem 5 mg/kg q8h compared with the no-treatment controls. However, the emergence of resistance was suppressed to below the limit of detection in rabbits receiving meropenem 30 mg/kg q8h s.c. (Fig. 2B and D), with an apparent inverted U relationship between meropenem drug exposure (measured by either AUC or %time > MIC) and the emergence of meropenem resistance.

A further cohort of rabbits received amikacin 3.33 mg/kg q8h administered intravenously (i.v.) (*n* = 6) or 5 mg/kg i.v. q8h (*n* = 6) in combination with meropenem 5 mg/kg s.c. q8h. Although this had minimal additional effect on the total bacterial population, the emergence of a meropenem-resistant population was suppressed compared with monotherapy (Fig. 1). The meropenem and amikacin pharmacokinetic data from the rabbit experiments are shown in Fig. S1 and S2.

### Pharmacokinetic-pharmacodynamic modeling

A PK-PD mathematical model was fitted to the pharmacokinetic and pharmacodynamic data from all rabbits receiving meropenem as monotherapy. The estimates for central tendency and dispersions for the model parameters are summarized in Table 1. The relatively high estimates of KPC indicate that a two-compartment structural model of the meropenem pharmacokinetics may not have been required.

**TABLE 1:** Median parameter estimates of the fitted PK-PD model^*a*^

Parameter	Median estimate	Credible interval (95%)
V (L)	2.15	1.51–2.31
CL (L/h)	3.18	2.78–4.14
Ka	11.72	6.10–17.41
KPC	72.60	45.381–89.03
KCP	2.69	1.44–7.15
Kgs	0.22	0.22–0.56
Kks	10.25	9.00–10.51
E50s (mg/L)	17.45	17.18–20.34
Kgr	0.11	0.11–0.20
Kkr	17.71	14.57–19.53
E50r (mg/L)	64.19	37.08–90.19
Hs	1.23	1.23–1.23
Hr	3.04	1.67–3.10
ICs (CFU/g)	8340	4619–18320
ICr (CFU/g)	1.61	1.11–2.37
POPmax (CFU/g)	40 × 10^9^	35 × 10^9^–41 × 10^9^

^*a*^ V is the volume of the central compartment; CL is the clearance of meropenem from the central compartment; Ka is the absorption constant; KCP and KPC are distribution constants for movement from the central to the peripheral compartments and vice versa; Kgs and Kgr are bacterial growth constants for the wild-type and resistant bacterial subpopulations; Kks and Kkr are bacterial kill constants for the same populations; POPmax represents the maximum bacterial density of the rabbit lung; E50s and E50r represent the concentration of meropenem that achieves 50% of the pharmacodynamic effect on the wild-type and resistant bacterial subpopulations; Hs and Hr are the Hill Equation constants for the respective populations; ICs and ICr are the initial conditions (i.e., at 0 hours) of the bacterial density for the wild-type and meropenem-resistant subpopulations.

**Fig 3:**
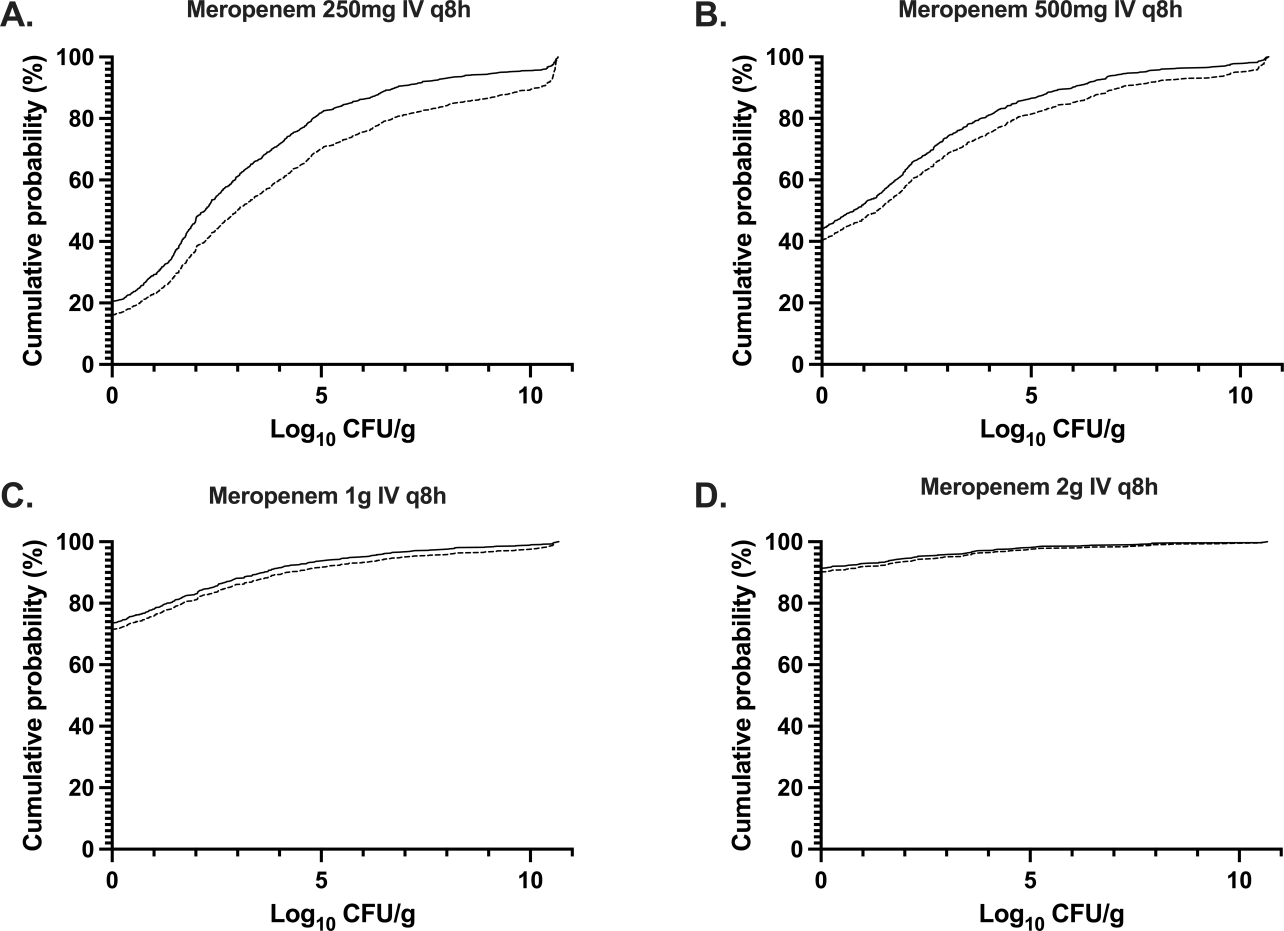
Cumulative probability distributions for attainment of different end-simulation bacterial densities in the lung of the population simulated by Monte Carlo simulation with the regimens meropenem 250 mg i.v. q8h (**A**), 500 mg i.v. q8h (**B**), 1 g i.v. q8h (**C**), and 2 g i.v. q8h (**D**). Cumulative probabilities are depicted by a continuous line for the total bacterial density and a dashed line for the meropenem-resistant bacterial densities.

To place the experimental results in a clinical context, a bridging study was performed using Monte Carlo simulation. Cohorts of simulated patients (*n* = 1,000/cohort) receiving 250 mg, 500 mg, 1 g, and 2 g IV q8h i.v. of meropenem were generated by using an existing population PK model of meropenem in critically ill patients (26). Adjustments were made for differences in the protein binding of meropenem in humans and rabbits. A 24-hour delay in initial treatment was used for all patients, and the effect was estimated after 96 hours (i.e., 72 hours of meropenem therapy). The cumulative probability distributions of the bacterial densities for both total bacteria and meropenem-resistant bacteria at the end of the simulation for all four simulated meropenem regimens are shown in Fig. 3. Simulated treatment with meropenem 500 mg or 1 g i.v. q8h failed to completely suppress the emergence of resistance with predicted rates of suppression of emergent resistant populations of 40.3% and 71.4%, respectively. In contrast, a regimen of meropenem 2 g i.v. q8h was predicted to have 90.2% complete suppression of emergent resistance. In all simulations where complete kill and resistance suppression were seen, the median log_10_ kill was 5.6 CFU/g.

### Histopathology

**Fig 4:**
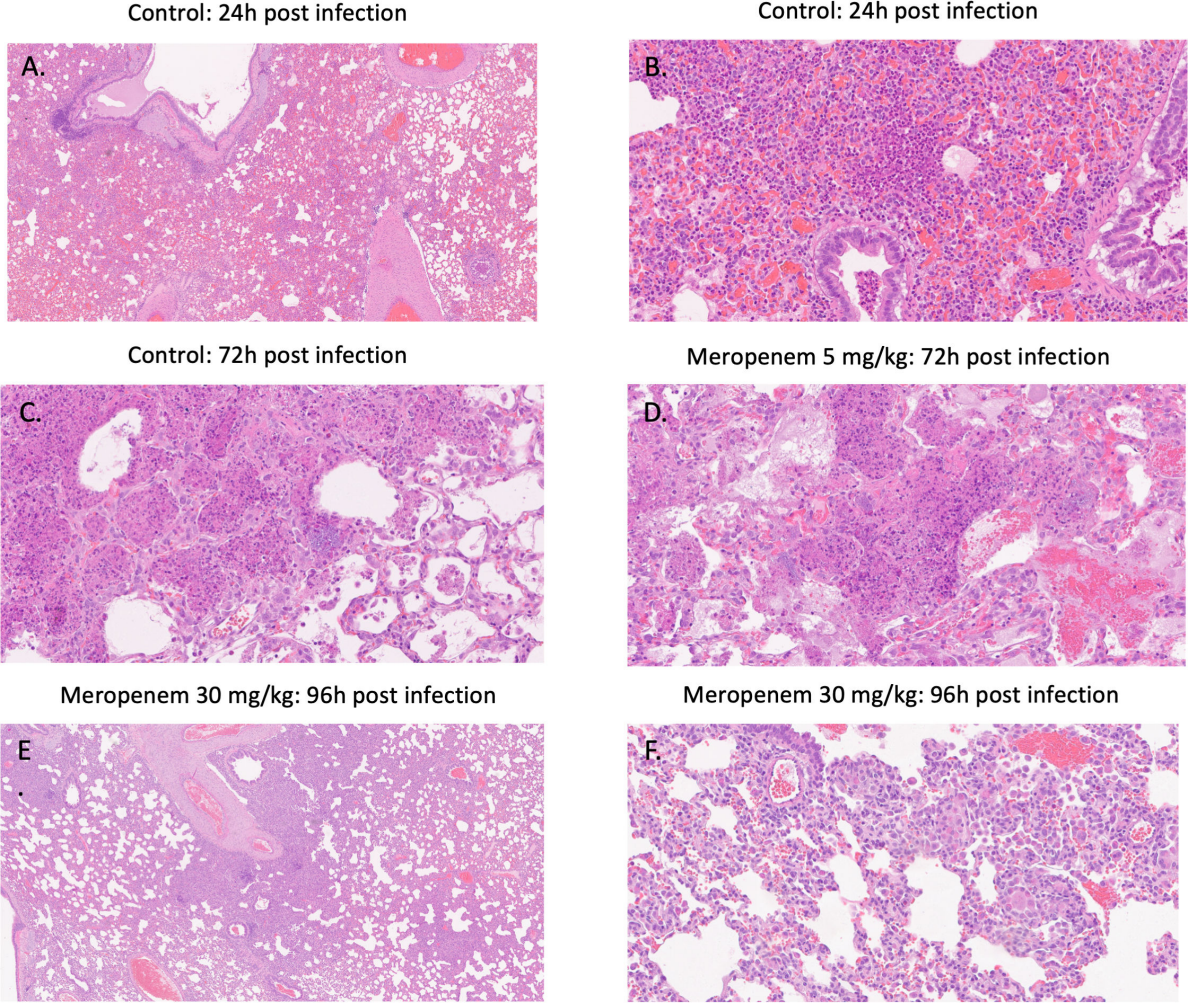
Representative histopathological sections of rabbit lung at 24 hours post infection (i.e., at time of treatment start) stained with HE, representing well established bacterial bronchopneumonia (panels A and B), at 72 hours post infection, with fulminant inflammation and infection in control and meropenem-treated rabbits (panels C and D, respectively) and at 96 hours post infection with resolving inflammation, infection, and tissue injury in a rabbit treated with meropenem 30 mg/kg s.c. q8h (panels E and F).

Findings consistent with *P. aeruginosa* bronchopneumonia were observed both macroscopically and microscopically with severity of infection increasing in a time-dependent fashion. Macroscopically, dark areas of hemorrhage were visible in all lobes and correlated with a time-dependent increase in lung weight in untreated controls and meropenem 5 mg/kg-treated animals (Table 2). A frothy discharge was also noted from the nasal cavity and trachea (post dissection). No abnormalities were observed macroscopically in meropenem 30 mg/kg-treated animals, with lungs averaging 17.6 g (SD 1.94 g). Microscopically, histopathological sections showed a well-established infection at initiation of drug treatment (Fig. 4) with marked multifocal heterophilic histiocytic inflammation and multifocal areas of consolidation due to dense inflammatory cell infiltrates with focal micro abscesses. At 72 hours post infection, 5 mg/kg/dose meropenem q8h s.c. was ineffective at limiting tissue damage and bacterial growth, with findings comparable with those of untreated controls. Conversely, at 96 hours post infection, meropenem 30 mg/kg/dose q8h s.c. was effective at limiting tissue damage, pulmonary inflammation, and visible bacteria within histopathological sections showing findings indicative of resolving inflammation, tissue injury and repair (Fig. 4).

**TABLE 2:** Mean rabbit lung weight from untreated control and the two meropenem monotherapy regimen arms. *n* = 2 for all groups

Group	Time post infection	Mean lung weight (g) (SD)
Untreated control	24 hours	24.2 (2.78)
Untreated control	72 hours	40.4 (9.31)
Meropenem—5 mg/kg s.c. q8h	72 hours	38.4 (3.04
Meropenem—30 mg/kg s.c. q8h	96 hours	17.6 (1.94)

### Interplay of MexAB-OprM and PDC-5 overexpression and selection of oprD mutants favor resistance adaptation in low-meropenem treatment dose

To better understand and comprehensively characterize the diversity of meropenem-resistant mutants in *P. aeruginosa*, we used a combination of whole-genome sequencing and quantitative real-time PCR (qPCR) strategies. The laboratory strain of *P. aeruginosa* ATCC 27853 has a large genome size (6,839,761 bp) that carries mutations at cytochrome oxidase C subunit I and zonula occludens toxin-containing protein when compared with National Center for Biotechnology Information reference genome (27). These mutations have no contribution toward resistance to meropenem. To identify single nucleotide polymorphisms (SNPs) and short insertions-deletions (indels), we mapped Illumina short reads from 12 lung isolates (*n* = 3 per group) to the reference genome of *P. aeruginosa* ATCC 27853. Using this approach, we found 9, 8, and 9 SNPs in 5 mg/kg, 30 mg/kg of meropenem, and 5 mg/kg meropenem plus 3.33 mg/kg amikacin combination-treated groups, respectively. Furthermore, we observed small numbers of chromosomal indels (*n* = 2, 1, and 4) in different treatment groups. In contrast, in the non-treatment group, we observed no SNPs or indels.

Interestingly, most of these SNPs and indels were in the *oprD* gene in all treatment groups, suggesting that loss of this outer membrane protein is the key first-step resistance mechanism in *P. aeruginosa*. We also observed mutations within *mexR* and *ampD* (encoding PBP4) transcriptional regulators, which were limited to only the 5 mg/kg treatment group (Table S1) (28–33). MexR is a known repressor of the MexAB-OprM efflux pump, and inactivation of *ampD* (PBP4) is associated with hyper-inducible AmpC β-lactamase expression in this pathogen (30, 34). However, these mutations were not present in rabbits receiving the meropenem 5 mg/kg s.c. with 3.33 mg/kg amikacin i.v. q8h combination treatment group.

**Fig 5:**
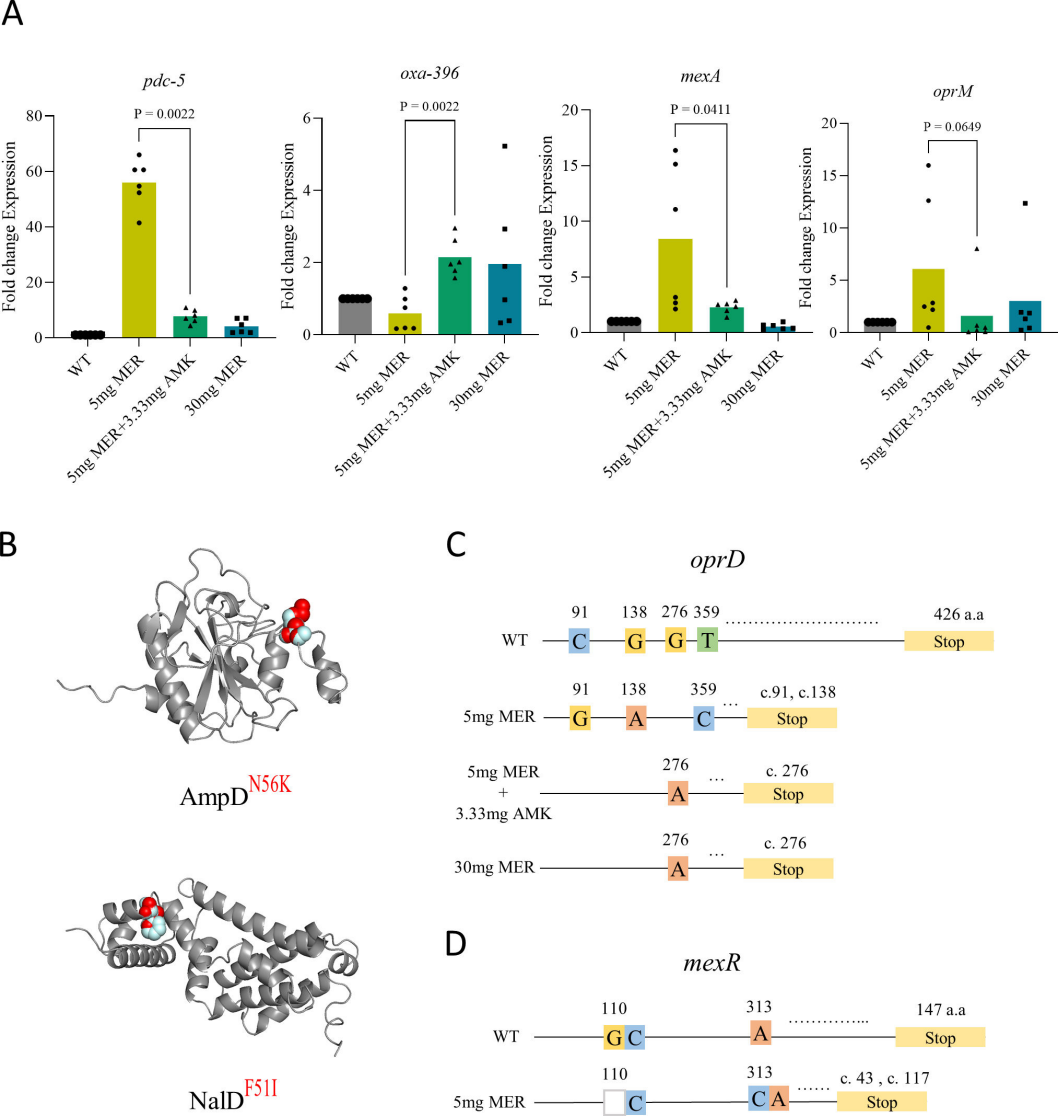
Meropenem treatment-associated dynamics of resistance mutations. (A) Normalized expression of gene *pdc-5*, *oxa-396*, *mexA*, and *oprM* with respect to 16S rRNA and *rpoB* genes. Each dot represents a single amplicon of different biological replicates (*n* = 6). Statistical analysis was carried out using Prism 9 (GraphPad, CA, USA). The two-tailed unpaired *t* test with Mann-Whitney post test was used for comparison between two groups. *P* values of <0.05 were considered statistically significant. (B) Non-synonymous substitution mapped on AmpD (AlphaFold ID: A0A3S0L9R0) and NalD (Protein Data Bank ID: 5DAJ). Gray shades represent monomers, and green/pink regions represent mutated residues. (C, D) Mutations identified in isolates obtained from different meropenem- and combination-treated group in (C) *oprD* and (D) *mexR* genes. Number represents nucleotide location in *oprD* and *mexR* genes, and colors are given to each box to highlight the base type. Blank box represents deletion of nucleotide base; yellow box represents stop codon; c.t. represents amino acid location at which chain terminal occurred due to SNPs and short indels.

The intricate pathway contributing to meropenem resistance in different treatment groups was further assessed using qPCR analysis with 16S rRNA and rpoD as housekeeping genes. The pulmonary *P. aeruginosa* mutant population recovered following treatment with meropenem 5 mg/kg s.c. was driven by overexpression of MexAB-OprM (8.42- ± 7.1-fold difference) and PDC-5- (55.94- ± 12.29-fold difference) type AmpC β-lactamase (Fig. 5). However, these resistance determinants were not overexpressed in rabbits receiving combination chemotherapy and high-dose meropenem compared with the wild type. The change in expression in MexR is associated with the frameshift mutations (p.His104Profs*12) and AmpD (N56K) transcriptional repressors. The slight upregulation of MexAB-OprD in the combination-treated group might be due to loss in function of another transcriptional repressor NalC. We also observed significant upregulation (~2-fold) of Oxa-396 genes in treatment groups, which lacked upregulation of the AmpC gene. To investigate the effect of the addition of amikacin with 5 mg/kg meropenem, the expression of aminoglycoside-specific RND-efflux pumps was analyzed. There was no observed fold change in the expression of MexXY or MexCD-OprM; however, MexEF-OprN displayed twofold upregulation in both 5 mg/kg meropenem monotherapy and combination therapy of 5 mg/kg meropenem and 3.33 mg/kg amikacin-treated rabbit groups.

## DISCUSSION

Treatment of HAP poses significant challenges with high rates of treatment failure for several reasons (35). Oropharyngeal colonization with resistant organisms acquired in the healthcare environment means that HAP pathogens have higher rates of resistance to broad-spectrum antibiotics (3). Additionally, the high inoculum burden of HAP infections above the inverse of typical mutation frequency value means that resistance commonly emerges during treatment, either due to *de novo* mutation or expansion of a pre-existing resistant subpopulation (16, 17, 19, 36). The high prevalence of *P. aeruginosa* in HAP, with intrinsic resistance to many antibiotics and a vast repertoire of adaptive mechanisms (with loss of the OprD porin, upregulation of efflux pumps [e.g., MexAB-OprM, MexXY-OprM, or MexCD-OprJ] and overexpression of AmpC being the most relevant clinically [37–39]), further limits successful treatment options (40). Optimization of antimicrobial dosing to successfully treat HAP and prevent emergence of resistance is clearly needed.

In the experimental work described here, we demonstrated a phenotypic inverted U relationship (41) between meropenem monotherapy dose and emergence of resistance (Fig. 2B), with co-administration of amikacin with 5 mg/kg q8h meropenem reducing the rate of emergent resistance compared with meropenem monotherapy (Fig. 1).

**Fig 6:**
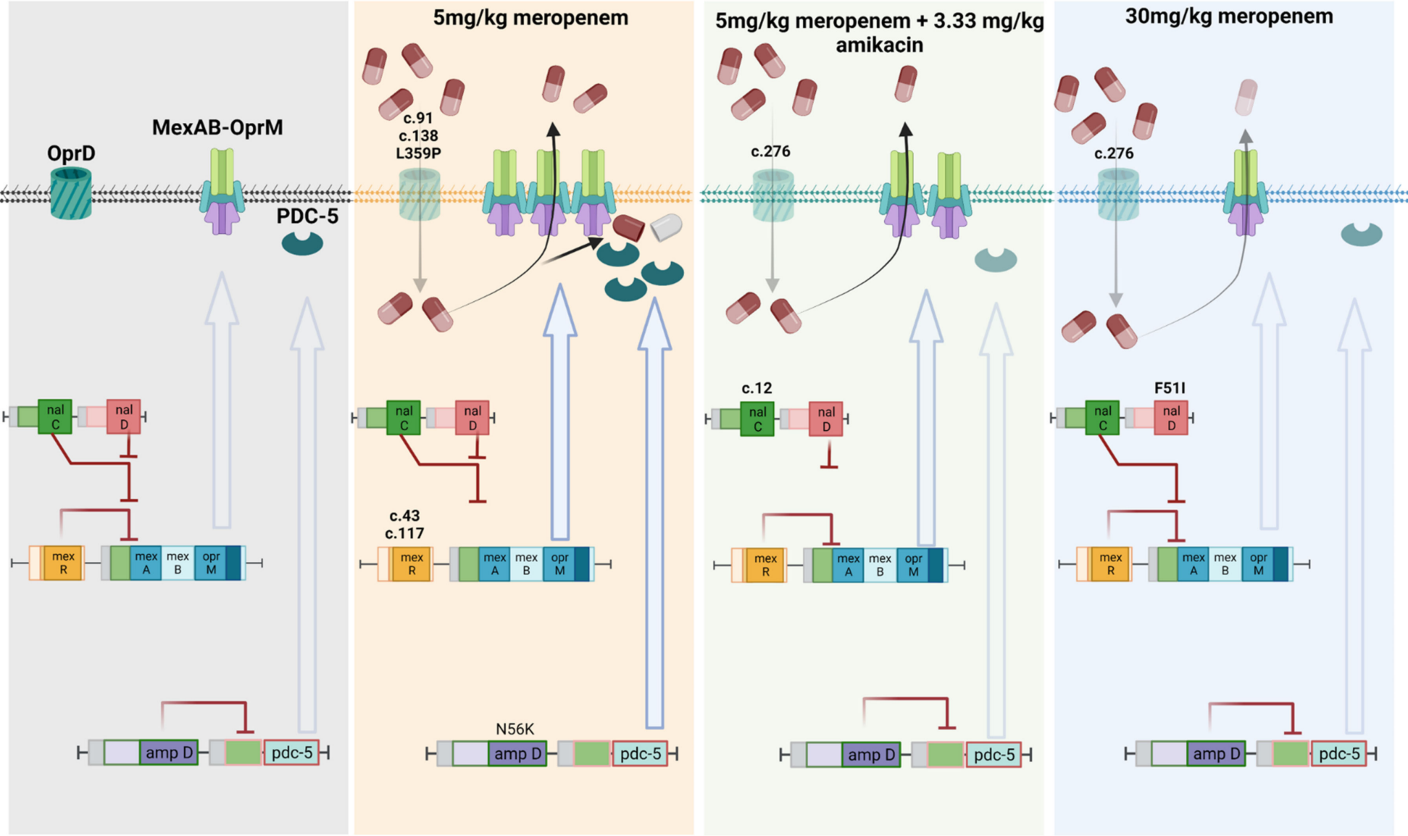
Schematic summary of the molecular mechanisms in *Pseudomonas aeruginosa* associated with the observed phenotypic changes in the rabbit model. OprD-associated mutations are common in all meropenem-resistant mutants. Administration of the meropenem regimen 5 mg/kg s.c. q8h led to a synergistic upregulation of adaptive resistance due to pdc-5 (N56K mutation of *ampD*) and mexAB efflux pump (frame shift mutations of cis-acting transcriptional repressor mexR). Co-administration of meropenem (5 mg/kg s.c. q8h) with amikacin (3.33 mg/kg i.v. q8h) displayed downregulation of *mexAB* (frame shift mutation of trans-acting transcriptional repressor *nalC*) and *pdc-5* seen in meropenem 5 mg/kg s.c. q8h monotherapy. All the adaptive mechanisms are downregulated in bacteria receiving the meropenem 30 mg/kg s.c. q8h regimen, with OprD disruption being the sole mechanism of resistance.

We characterized the nature of this resistance genotypically. Treatment with meropenem 5 mg/kg i.v. q8h resulted in a high-fitness resistant phenotype where a well-choreographed network exists between the MexAB-OprM RND-efflux pump, PDC-5-type AmpC β-lactamase, and OprD porin (Fig. 6). We observed frameshift mutations in the 5 mg/kg meropenem group in the protein coding sequence of mexR, one of the transcriptional repressors for the MexAB-OprM efflux system (42), which resulted in its early transcriptional termination. Similarly, a point mutation on the DNA binding helix-turn-helix motif was observed in the *ampD* transcriptional repressor of AmpC β-lactamase (43, 44). The emergence of resistant mutants, in general, was reduced in frequency during treatment with meropenem 5 mg/kg meropenem with amikacin 3.33 mg/kg amikacin in combination and 30 mg/kg meropenem monotherapy. However, where they emerged, they were mainly facilitated by selection of low-fitness *oprD* mutants.

This experimental work therefore suggests two routes for mitigating treatment failure and counterselection of resistance during treatment of HAP caused by wild-type *P. aeruginosa* with meropenem: (i) intensification of regimen to move above the inverted U relationship of emergent resistance and (ii) use of combination therapy to prevent the emergence of resistance. The latter is in keeping with other *in vitro* and *in vivo* experimental work examining the effect of β-lactam/aminoglycoside combinations on antimicrobial resistance in *P. aeruginosa* (45–48).

The simulated effects of meropenem monotherapy using human drug exposures demonstrate increasing rates of emergence of resistance suppression with increasing dose (Fig. 3), with maximal suppression on a population level with the highest licensed dose of meropenem (meropenem i.v. 2 g q8h). Correspondingly, this indicates that this regimen may be required in the treatment of HAP with meropenem monotherapy for maximal treatment success and prevention of the emergence of resistance.

There are some limitations to our conclusions. First, the experimental work was performed using meropenem-susceptible wild-type *P. aeruginosa* only. Alternative strategies will likely be needed for meropenem non-susceptible *P. aeruginosa* strains. Secondly, the simulations predicting human exposure are subject to some assumptions, most importantly that the distribution of meropenem (particularly into the lung tissue and epithelial lung fluid) is equivalent in rabbits and humans.

Beyond the implications for use of meropenem, this work has implications for wider drug development. Traditional *in vivo* infection models (of HAP or otherwise) assess only for bacterial kill pharmacodynamics, even for recently developed antimicrobial agents (49–54). This helps define a regimen that achieves the PK-PD target for bacterial kill at a population level, often optimized to minimize the material cost of the drug (55, 56). However, the PK-PD targets for prevention of the emergence of resistance are, when determined (typically in HFIM experiments), usually different to the target for bacterial kill (as illustrated with piperacillin-tazobactam and fosfomycin—see, for example, references 57, 58) and do not normally inform the eventual licensed clinical regimen (55). This means that while the final clinical regimens may successfully treat susceptible infections, there is an ongoing and realized risk of emergent resistance while on therapy due to failure to meet the resistance PK-PD target. This is especially the case for high-inoculum infections such as HAP, as described earlier.

This suboptimal dosing for prevention of emergent resistance is not only a problem for the treated individual in whom this emergent resistance occurs while on therapy. Horizontal transmission of resistant isolates to the environment and distant others means that the implications of suboptimal dosing are significant. If antimicrobial agents (both novel and currently licensed) are to be used sustainably (i.e., without the emergence of resistance on a population level), antimicrobial regimens need to be optimized to achieve the pharmacodynamic target for prevention of resistance, whether by regimen intensification, co-administration with other agents (where specific combinations have been demonstrated to prevent emergence of resistance), or other strategies, in addition to the pharmacodynamic target for bacterial kill.

With parallel phenotypic and genotypic characterization of the pharmacodynamics of emergent resistance, our experimental platform allows insight into the mechanisms of resistance development on therapy and clinical failure. From this, strategies can be explored and developed to mitigate the emergence of resistance in the clinical regimen. This experimental methodology, therefore, represents an innovative non-clinical platform to inform sustainable dose optimization of antimicrobials both in terms of bacterial kill and in terms of prevention of the emergence of resistance for pneumonia.

While the HFIM has been used for modeling HAP (including the emergence of resistance) previously (19, 20, 59, 60), this is, to our knowledge, the first rabbit HAP model to characterize resistance alongside bacterial kill. The principal benefit of using the rabbit model over the HFIM is that the rabbit model of pneumonia is a better mimic of human anatomy, physiology, and pathogenesis. The main disadvantages of this model is the infrastructure and skillbase required to conduct these experiments safely and effectively and the number of different drug regimens that can be feasibly studied.

Despite these limitations, this model represents an innovative platform permitting much needed characterization of the pharmacodynamics of emergent resistance alongside typical *in vivo* bactericidal pharmacodynamic characterization. This allows development of mitigation strategies for antimicrobial use (whether in novel drug development or repurposing of older drugs) to allow sustainable use of antimicrobials with minimized emergent resistance, which are fundamentally required in the wider fight against anti-microbial resistance.

## MATERIALS AND METHODS

### Rabbit model of nosocomial pneumonia

All laboratory animal experiments were conducted under UK Home Office project License PP3585942 and approved by the University of Liverpool Animal Welfare Ethics Review Board. A neutropenic rabbit model of nosocomial pneumonia was developed. Male New Zealand White (NZW) rabbits (Envigo, UK) weighing 2.55–3.12 kg at the time of experimentation were used for all experiments. Rabbits were rendered neutropenic by administration of cytarabine (Accord Healthcare Ltd., North Harrow, UK) IV on a q24h regimen starting 24 hours prior to infection.

A target inoculum of 5 × 10^7^ CFU/mL *Pseudomonas aeruginosa* ATCC 27853 was prepared by emulsifying a single colony from MH agar plates into MH broth and placing in a shaking incubator at 37°C. After 4 hours, the bacterial suspension was placed in a centrifuge for 5 min at 1,500 × *g*. The supernatant was removed, and the bacterial pellet as adjusted to the correct density on the spectrophotometer using sterile phosphate-buffered saline (PBS). The exact inoculum was verified with quantitative culture. Rabbits were inoculated while anesthetized (medetomidine [Vetoquinol, UK], 0.2 mg/kg administered intramuscularly [i.m.] plus ketamine [Chanelle Pharma, UK] 15 mg/kg i.m.) by inoculating 2 mL of the 5 × 10^7^ CFU/mL suspension into the endobronchial tree. Animals were serially sacrificed at planned time points unless they exhibited prespecified humane endpoints that mandated immediate sacrifice to minimize suffering.

### *In vivo* PK-PD studies

Treatment with meropenem (Venus Pharma GmbH, Germany) was initiated 24 hours post inoculation. Meropenem monotherapy dosages of 5 and 30 mg/kg/dose or in combination with amikacin at dosages of 3.33 or 5 mg/kg were administered q8h as either subcutaneous injection (meropenem) or an i.v. push via the marginal ear vein (amikacin). Blood samples of approximately 0.5 mL were taken from the marginal ear vein of each rabbit for PK analyses. Samples were collected pre-dose (0 h) and at 15 min, 30 min, 1 h, 2 h, 4 h, 6 h and 8 h. Whole blood was placed into Eppendorf tubes containing approximately 10 mL heparin (Wockhardt, UK) and centrifuged. Plasma was eluted and stored at −80°C for subsequent bioanalysis.

Lungs were removed and carefully dissected into individual lobes. Each lobe was placed in 5 mL sterile PBS, weighed, and homogenized before being serially diluted and plated onto drug-free and drug-containing (3 mg/L meropenem) Mueller Hinton agar (MHA) (Southern Group Laboratory, Corby, UK) for quantitative analysis. Isolates taken from the drug-containing MHA plates in the treatment-receiving arms and drug-free MHA plates in the untreated control groups were selected for RT-PCR and whole-genome sequencing.

### Bioanalytical method

Meropenem and amikacin were extracted from rabbit plasma and analyzed using the following processes.

#### Meropenem

The internal standard, [^2^H_6_] meropenem (Alsachim, France), was prepared in acetonitrile (5 mg/L, Fisher Scientific UK), of which 250 µL was added to a 96-well protein precipitation plate (Phenomenex, Cheshire, UK). Fifty microliters of the sample, blanks, calibrators in the range 0.1–50 mg/L, and quality controls (0.75, 7.5, and 37.5 mg/L) was mixed with the internal standard, and the plate was placed on an orbital shaker for 2 min. Liquid was drawn through the protein precipitation plate into a collection plate using a positive pressure manifold. Water and 0.1% formic acid (750 µL) were added to each well. The plate was sealed and placed onto an orbital shaker for 5 min before being transferred to the autosampler for analysis by liquid chromatography with tandem mass spectrometry (LC-MS-MS).

LC-MS-MS analysis was performed using an Agilent 1290 Infinity HPLC coupled to an Agilent 6420 triple quadrupole mass spectrometer fitted with an electrospray source. The LC-MS system was controlled using Agilent MassHunter Data Acquisition software (Ver B.06.00). Analytes were injected (2 µL) onto a Phenomenex Kinetex C18 100 Å column (2.1 mm × 50 mm, 2.6 µm, and 40°C) and separated over a 5.5-min gradient using a mixture of solvents A and B. Solvent A was LC-MS-grade water with 0.1% (vol/vol) formic acid. Solvent B was HPLC-grade acetonitrile with 0.1% (vol/vol) formic acid. Separations were performed by applying a linear gradient of 2%–98% solvent B over 4 min at 0.6 mL/min followed by an equilibration step (1.5 min at 2% solvent B).

The mass spectrometer was operated in positive ion mode using a Multiple Reaction Monitoring (MRM) method. Following an optimization process, the following mass transitions and collision energies were used for the analysis: 384.16 > 141.2 (Ce 12 eV) and 390.2 > 147.1 (Ce 16 eV). The mass spectrometer conditions were as follows: capillary voltage of 3.5 kV, fragmentor voltage of 100 V, source gas temperature of 350°C, and gas flow of 11 L/min. The resultant data were processed using Agilent Mass Hunter Quantitative Analysis (Ver B.05.02).

The lower limit of quantification (LLQ) of meropenem was 0.1 mg/L in rabbit plasma. The inter- and intra-day %CV was <15%, and the analyte was stable in all conditions described above. Accuracy was assessed across three different quality control (QC) levels during validation with a range of −1.16%–8.56%.

#### Amikacin

The internal standard, [^2^H_5_] amikacin (Alsachim, France), was prepared in acetonitrile + 5% trichloroacetic acid (10 mg/L, Fisher Scientific, UK), and 150 μL was added to a 96-well protein precipitation plate (Phenomenex, Cheshire, UK). Fifty microliters each of plasma sample, blanks, calibrators in the range 0.1–25 mg/L, and quality controls (0.75, 3.75, and 12.5 mg/L) was mixed with the internal standard on an plate shaker for 5 min at 800 rpm. Liquid was drawn through the protein precipitation plate into a collection plate using a positive pressure manifold. Samples were then evaporated to dryness using nitrogen (40 L/min for 40 min at 40°C). Samples were then reconstituted in with 80:20 water:MeOH + 0.1% heptafluorobutyric acid (HFBA). The plate was sealed and placed onto an orbital shaker for 10 min at 800 rpm before being transferred to the autosampler for analysis by LC-MS-MS.

LC-MS-MS analysis was carried out using an Agilent 1290 Infinity HPLC coupled to an Agilent 6420 triple quadrupole mass spectrometer fitted with an electrospray source. The LC-MS system was controlled using Agilent MassHunter Data Acquisition software (Ver B.06.00). Analytes were injected (5 μL) onto a Discovery HS C18 HPLC Column (3 µm 5 cm × 2.1mm) and separated over a 3.5-min gradient using a mixture of solvents A and B. Solvent A was LC-MS-grade water with 0.1% (vol/vol) HFBA. Solvent B was LC-MS-grade acetonitrile with 0.1% (vol/vol) HFBA. Separations were performed by applying a gradient of 2%–98% solvent B over 2 min at 0.5 mL/min followed by an equilibration step (0.5 min at 2% solvent B).

The mass spectrometer was operated in positive ion mode using a MRM method. Following an optimization process, the following mass transitions and collision energies were used for the analysis: 586.4 > 163.2 (Ce 30 eV) and 591.3 > 163.2 (Ce 30 eV). The mass spectrometer conditions were as follows: capillary voltage of 3.5 kV, fragmentor voltage of 100 V, source gas temperature of 350°C, and gas flow of 11 L/min. The resultant data were processed using Agilent Mass Hunter Quantitative Analysis (Ver B.05.02).

The LLQ of amikacin was 0.1 mg/L in rabbit plasma. The mean inter- and intra-day %CV was 2.65% and 2.54%, respectively, and the analyte was stable in all conditions described above. Accuracy was assessed across three different QC levels during validation with a range of −1.05%–6.47%.

### Histopathology

The progression of infection at a microscopic level was assessed by placing lung tissue from each rabbit in 10% neutral-buffered formalin (Sigma-Aldrich, St Louis, USA). Tissues were embedded in paraffin and sectioned using standard protocols. Sections were stained with hematoxylin and eosin (HE) and Gram stain using standard approaches and evaluated by a board-certified veterinary pathologist.

### PK-PD modeling

Experimental pharmacokinetic and pharmacodynamic data were fitted using a non-parametric adaptive grid algorithm with the program Pmetrics (61) to a mathematical model with the following structural model:


$$XP(1)=-Ka{\;}^{*}\;X(1)$$

$$\begin{array}{r} XP(2)=Ka{\;}^{*}\;X(1)-(CL/V){\;}^{*}\;X(2)-KCP{\;}^{*}\;X(2)+KPC{\;}^{*}\;X(3) \end{array}$$

$$XP(3)=KCP{\;}^{*}\;X(2)-KPC{\;}^{*}\;X(3)$$

$$\begin{array}{rl} & XP(4)=(Kgs{\;}^{*}\;X(4){\;}^{*}\;(1-((X(4)+X(5))/POPmax)))-\\ & (Kks{\;}^{*}\;X(4){\;}^{*}\;((X(2)/V){\;}^{**}\;Hs)/((E50s{\;}^{**}Hs)+((X(2)/V){\;}^{**}Hs))) \end{array}$$

$$\begin{array}{rl} & XP(5)=(Kgr{\;}^{*}\;X(5){\;}^{*}\;(1-((X(4)+X(5))/POPmax)))-\\ & (Kks{\;}^{*}\;X(5){\;}^{*}\;((X(2)/V){\;}^{**}\;Hr)/((E50r{\;}^{**}Hr)+((X(2)/V){\;}^{**}Hr))) \end{array}$$

XP(1)–XP(5) are differential equations describing the changes in X(1) – X(5). X(1), X(2), and X(3) represent amounts of meropenem in the absorption, central, and peripheral compartments, respectively. X(4) and X(5) describe the pharmacodynamics of the meropenem-susceptible and resistant bacterial populations in the rabbit lung. V is the volume of the central compartment; CL is the clearance of meropenem from the central compartment; Ka is the absorption constant from the subcutaneous injection site to the plasma; KCP and KPC are first-order rate constants describing the movement of mass to and from the central and peripheral compartments; Kgs and Kgr are bacterial growth constants for the susceptible and resistant bacterial subpopulations; Kks and Kkr are bacterial kill constants for the same populations; POPmax represents the maximum bacterial density the rabbit lung; E50s and E50r represent the concentration of meropenem that achieves 50% of the pharmacodynamic effect in the susceptible and resistant bacterial subpopulations; Hs and Hr are the Hill Equation constants for the respective populations. ICs and ICr are the initial conditions of the bacterial density for the susceptible and meropenem resistant subpopulations.

There were three model outputs:


Y(1)=X(2) / V

Y(2)=X(4)+X(5)

Y(3)=X(5)

These outputs correspond to meropenem concentration (mg/L) [Y(1)], total bacterial concentration (CFU/mL) [Y(2)] and meropenem-resistant subpopulation concentration (CFU/mL) [Y(3)].

As a bridging study, simulations were performed using ADAPT (62) using human meropenem PK from a previously published model (26). Monte Carlo simulations were performed using parameter and covariance values from this model for the PK parameters and from our fitted PD parameters from the rabbit model. Although the published model was a three-compartment model, including an epithelial lining fluid (ELF) compartment, we linked the effect to the meropenem concentrations in the central compartment. We therefore make the assumption that meropenem distribution to the ELF occurs at a comparable rate and extent in rabbits and humans. Within the model, multiple traps were used to prevent negative values for both drug amount and bacterial densities. For bacterial densities, these traps caused any zero or negative value to revert to a value of 0.001 log_10_ CFU/g to avoid log transform errors. Additionally, a floor of 1 × 10^0^ CFU/g was applied to the simulated bacterial density results, to replicate the experimental lower limit of detection. Differential species’ meropenem protein binding was accounted by incorporation of a protein binding scalar. Two different experimental comparisons of meropenem binding in humans and rabbits exist in the literature (63, 64). As a sensitivity analysis, we performed the simulation with both protein binding parameters, but this made little difference to the simulation results. We therefore opted to use the protein binding parameters of 21.2% in rabbits and 13% in humans from the peer-reviewed source (63).

### Illumina sequence analysis

Briefly, base calling and de-multiplexing of indexed reads were performed by CASAVA version 1.8.2 (Illumina, San Diego, USA) to produce samples in FASTQ format. The raw FASTQ files were trimmed to remove Illumina adapter sequences using Cutadapt version 1.2.1 (65). The option “-O 3” was set, so the 3′ end of any reads which matched the adapter sequence over at least 3 bp was trimmed off. The reads were further trimmed to remove low-quality bases, using Sickle version 1.200 with a minimum window quality score of 20. After trimming, reads shorter than 20 bp were removed.

### Variant calling

To identify pre-existing resistance mutations that were present at the start of the infection, forward and reverse reads from all samples were mapped to the assembly using BWA mem version 0.7.5a (66) with default parameters. To retain only confidently aligned reads, alignments were filtered to remove reads with a mapping quality lower than 10, which equates to a 10% chance that the read was derived from another genomic location. To avoid duplicate reads arising from PCR amplification, read duplicates were identified and filtered to retain only a single representative, using the Picard “MarkDuplicates” tool, version 1.85 (http://picard.sourceforge.net/). Variant detection was performed using the GATK (67, 68). SNPs, and small indels were identified in the same analysis. Single-sample genomic VCFs (GVCFs) were imported into GenomicsDB before joint genotyping using the GATK GenotypeGVCFs tool. Variants were filtered using the GATK VariantFiltration tool (67) following assessment GATK filtering cutoff, i.e., In the resulting VCF files, SNPS with either QD < 2, FS > 60, MQ < 40, SOR > 3, QUAL < 30, MQRankSum < −12.5, or ReadPosRankSum < −8 and indels with QD < 2, FS > 200, QUAL < 30, and ReadPosRankSum < −20 were flagged according to the reason that they failed filtering. Filtered files were eventually annotated with SnpEff v4.2 (69), and SNPs and InDels located in a set of genes known to be involved in *P. aeruginosa* chromosomal antibiotic resistance were extracted.

### Real-time PCR

Briefly, the total RNA was isolated using TRI reagent (Sigma-Aldrich, USA) after adjusting the respective optical density at 600 nm (OD_600_) of each bacterial inoculum to 0.8. Following DNase treatment, 1 µg of DNA-free RNA from different treatment samples was used to prepare cDNA using the M-MuLV reverse transcriptase kit (New England Biolabs, Ipswich, USA). Real-time PCR was performed with the SYBR green master mix (Applied Biosystems, Waltham, USA) following the manufacturer’s instructions. Measurements were performed using the QuantStudio 6 real-time PCR system (Applied Biosystems, Waltham, USA) with the following conditions: 95°C for 10 min, 40 cycles of 95°C for 15 s, and 60°C for 1 min and a final dissociation cycle of 95°C for 2 min, 60°C for 15 s, and 95°C for 15 s. Relative gene expression was calculated and normalized by the ΔΔCT method using *P. aeruginosa* 16S rRNA as the reference gene. Primers used for real-time PCR are given in Table S1 (supplementary material).

## Data Availability

The software packages used to model and simulate the pharmacokinetic/pharmacodynamic data, Pmetrics and ADAPT, are freely available at https://www.lapk.org/software.php and https://bmsr.usc.edu/software/adapt/, respectively. The genomic data produced and analyzed in the work described in this paper can be accessed at the URLs https://cgr.liv.ac.uk/illum/LIMS30831_21f0ce38c849f10a/ (for trimmed fastq files) and https://cgr.liv.ac.uk/illum/LIMS30381Results_da7ecc07792287cf/ (for vcf files).

## SUPPLEMENTAL MATERIAL

Supplemental tables and figures mbio.03165-23-s0001.docx Figures S1 and S2 and Tables S1 and S2.

ASM does not own the copyrights to Supplemental Material that may be linked to, or accessed through, an article. The authors have granted ASM a non-exclusive, world-wide license to publish the Supplemental Material files. Please contact the corresponding author directly for reuse.
