# Supplementary material for: Molecular pharmacodynamics of meropenem for nosocomial pneumonia caused by Pseudomonas aeruginosa
Source: mBio. 2024 Jan 18;15(2):e03165-23. doi: 10.1128/mbio.03165-23 (PMC10865990; doi:10.1128/mbio.03165-23)
Supplement: Supplemental tables and figures — Figures S1 and S2 and Tables S1 and S2. [file mbio.03165-23-s0001.docx]

**Molecular pharmacodynamics of meropenem for nosocomial pneumonia caused by *Pseudomonas aeruginosa***

**Supplementary Material**

**Supplementary Tables**

| **Gene** | **Gene ID** | **Mutation site** | **Mutation** | **Type** | **Function** | **Ref** | |
| --- | --- | --- | --- | --- | --- | --- | --- |
| **Isolates treated with 5mg meropenem** | | | | | | | |
| oprD | NP446_RS22285 | 4720604 | C-G | SNP | Porin for carbapenem influx | | (1) |
| oprD | NP446_RS22285 | 4720745 | G-A | SNP | - | | (1) |
| oprD | NP446_RS22285 | 4721407 | T-C | SNP | - | | (1) |
| ampD | NP446_RS25910 | 5487757 | A-C | SNP | Transcriptional repressor of AmpC β-lactamase | | (2) |
| mexR | NP446_RS02205 | 481104 | T-TG | INDEL | Transcriptional repressor of MexAB-oprM efflux pump | | (3) |
| mexR | NP446_RS02205 | 481306 | GC-G | INDEL | - | | (3) |
| Isolates treated with 5mg meropenem and 3.33mg amikacin | | | | | | | |
| oprD | NP446_RS22285 | 4721161 | G-A | SNP | - | | (1) |
| nalC | NP446_RS06070 | 1274401 | GGCGTTGCCTGCC-G | INDEL | Transcriptional repressor of MexAB-oprM efflux pump | | (4) |
| Isolates treated with 30mg meropenem | | | | | | | |
| nalD | NP446_RS07125 | 1477005 | A-T | SNP | Transcriptional repressor of MexAB-oprM efflux pump | | (5) |
| oprD | NP446_RS22285 | 4721161 | G-A | SNP | - | | (1) |
| spot | NP446_30990 | 6580143 | G-GATGGCC | INDEL | ppGpp accumulation in stringent condition | | (6) |

**Table S1. List of mutations in *P. aeruginosa* isolates treated with different doses of meropenem**

| **Gene/primers** | **Direction** | **Sequence (5’→3’)** |
| --- | --- | --- |
| 16S rRNA | forward | GGGGTAAAGCGCGCGTAG |
| 16s rRNA | reverse | GCCACTGGTGTTCCTTCCTA |
| RPO | forward | GGGCTGTCTCGAATACGTTGA |
| RPO | reverse | ACCTGCCGGAGGATATTTCC |
| PDC-5 | forward | TGGCATCGAGCGCCTGCG |
| PDC-5 | reverse | CCCGCTACGGGTCGGTCC |
| OXA-396 | forward | GCGAACAGCCTGATCGG |
| OXA-396 | reverse | GGTACGTTCGATGCCTTGAT |
| MexA | forward | CCAGCGCTACAAGCTGCTGGT |
| MexA | reverse | TCGGCGACAGCACCTTGGTGT |
| OprM | forward | TGGGAACTCGATCTCTTCGGC |
| OprM | reverse | GCCGAAGAGATCGAGTTCCCA |
| MexC | forward | GGAAGAGCGACAGGAGGC |
| MexC | reverse | CTGCACCGTCAGGCCCTC |
| MexE | forward | TACTGGTCCTGAGCGCCT |
| MexE | reverse | TCAGCGGTTGTTCGATGA |

**Table S2 –** Details of primers used in methodology.

**Supplementary figures**

**
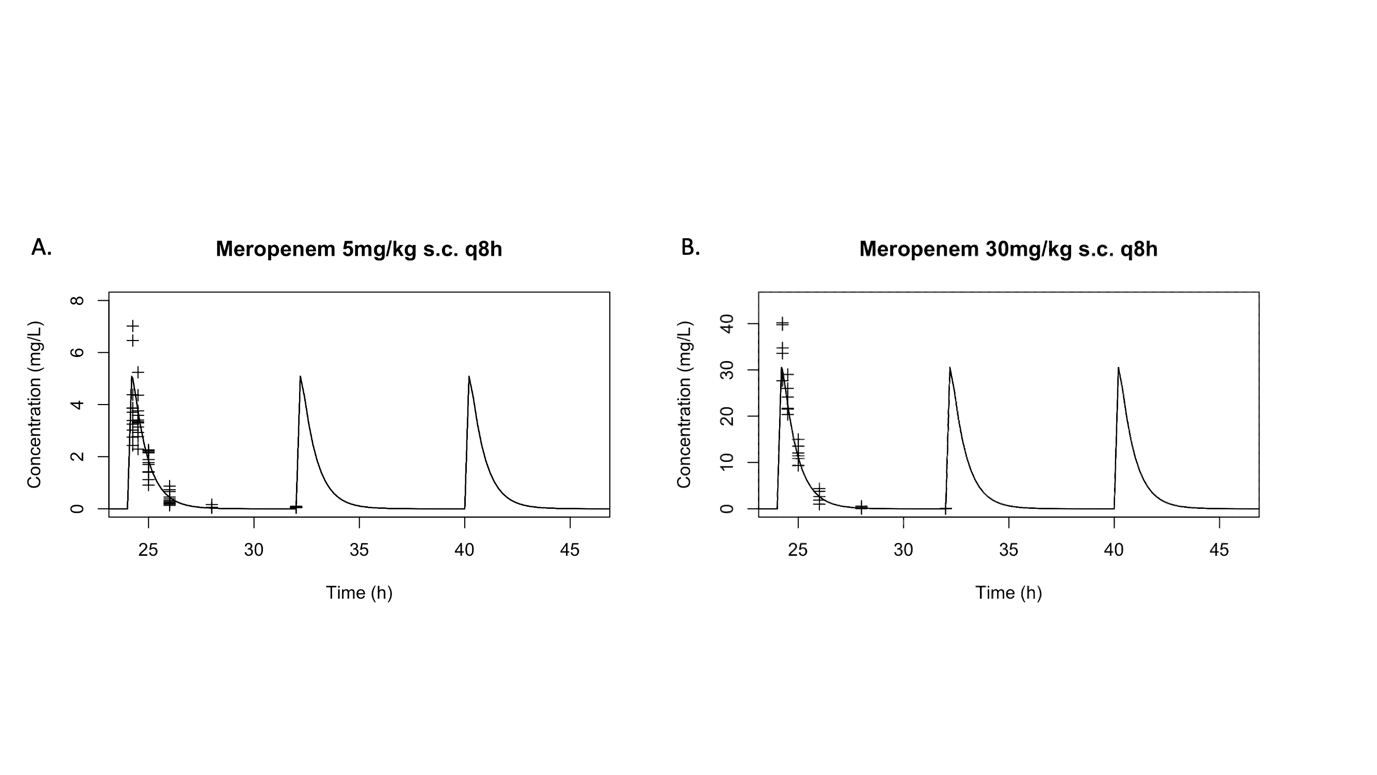
**

**Figure S1 –** Measured meropenem rabbit plasma concentrations (crosses) and modelled rabbit plasma time-concentration profile derived from the fitted PK-PD model (solid line) for experimental regimens of meropenem 5mg/kg s.c. q8h (A) and meropenem 30mg/kg s.c. q8h (B) for the period 24-48h after experiment started and 0-24h after administration of meropenem started.

**
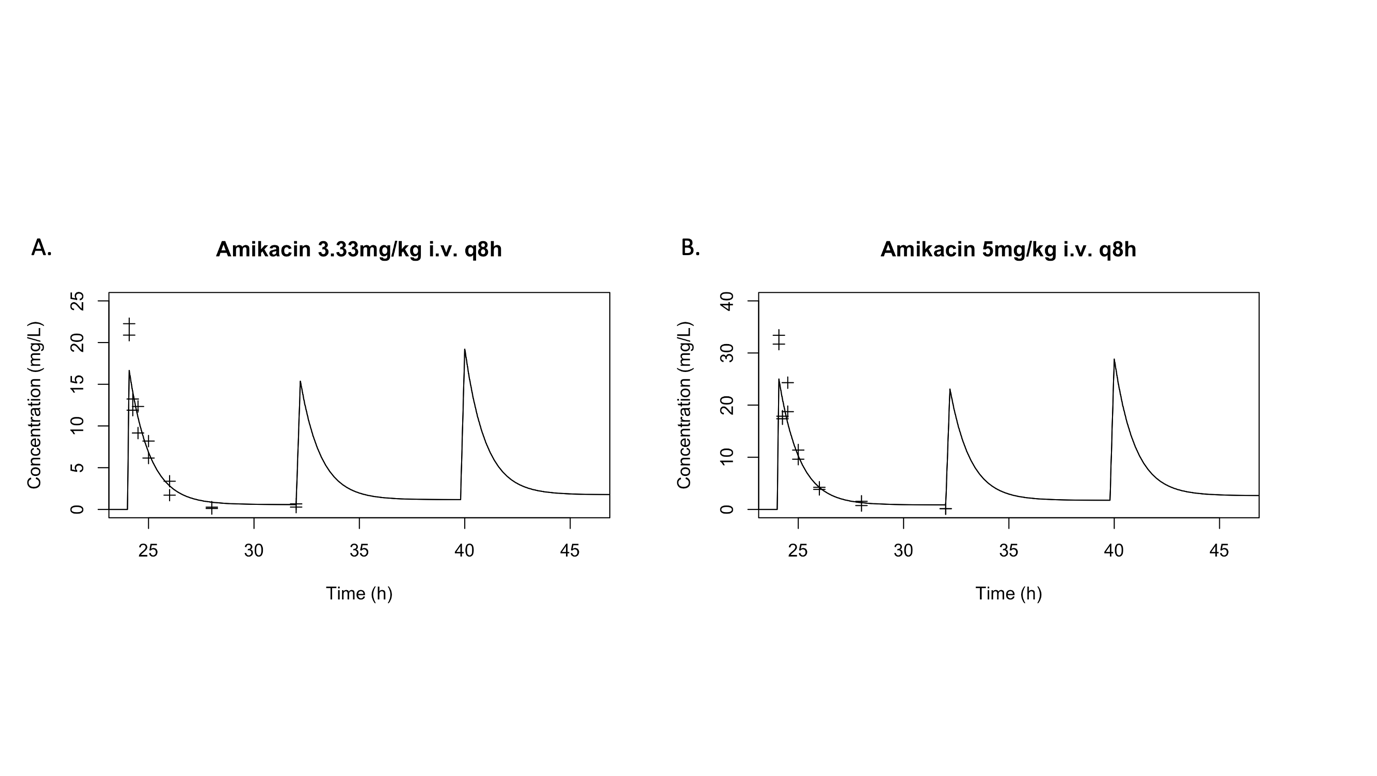
**

**Figure S2 –** Measured amikacin rabbit plasma concentration (crosses) and modelled rabbit plasma time-concentration profile derived from a fitted amikacin PK model (solid line) for experimental regimens of (A) amikacin 3.33mg/kg i.v. q8h and (B) amikacin 5mg/kg i.v. q8h for the period 24-48h after experiment started and 0-24h after administration of meropenem started.

**References**

1. Ocampo-Sosa AA, Cabot G, Rodríguez C, Roman E, Tubau F, Macia MD, et al. Alterations of OprD in Carbapenem-Intermediate and -Susceptible Strains of Pseudomonas aeruginosa Isolated from Patients with Bacteremia in a Spanish Multicenter Study. Antimicrob Agents Chemother. 2012 Apr;56(4):1703–13.

2. Langaee TY, Gagnon L, Huletsky A. Inactivation of the ampD Gene in Pseudomonas aeruginosa Leads to Moderate-Basal-Level and Hyperinducible AmpC β-Lactamase Expression. Antimicrob Agents Chemother. 2000 Mar;44(3):583–9.

3. Adewoye L, Sutherland A, Srikumar R, Poole K. The MexR Repressor of the mexAB-oprM Multidrug Efflux Operon in Pseudomonas aeruginosa: Characterization of Mutations Compromising Activity. J Bacteriol. 2002 Aug;184(15):4308–12.

4. Braz VS, Furlan JPR, Fernandes AFT, Stehling EG. Mutations in NalC induce MexAB-OprM overexpression resulting in high level of aztreonam resistance in environmental isolates of Pseudomonas aeruginosa. FEMS Microbiol Lett. 2016 Aug;363(16):fnw166.

5. Sobel ML, Hocquet D, Cao L, Plesiat P, Poole K. Mutations in PA3574 (nalD) Lead to Increased MexAB-OprM Expression and Multidrug Resistance in Laboratory and Clinical Isolates of Pseudomonas aeruginosa. Antimicrob Agents Chemother. 2005 May;49(5):1782–6.

6. Viducic D, Ono T, Murakami K, Susilowati H, Kayama S, Hirota K, et al. Functional analysis of spoT, relA and dksA genes on quinolone tolerance in Pseudomonas aeruginosa under nongrowing condition. Microbiol Immunol. 2006;50(4):349–57.
